# Membrane Protein Structures in Lipid Bilayers; Small-Angle Neutron Scattering With Contrast-Matched Bicontinuous Cubic Phases

**DOI:** 10.3389/fchem.2020.619470

**Published:** 2021-02-09

**Authors:** Charlotte E. Conn, Liliana de Campo, Andrew E. Whitten, Christopher J. Garvey, Anwen M. Krause-Heuer, Leonie van 't Hag

**Affiliations:** ^1^School of Science, STEM College, RMIT University, Melbourne, VIC, Australia; ^2^Australian Centre for Neutron Scattering, Australian Nuclear Science and Technology Organisation, Lucas Heights, NSW, Australia; ^3^Lund Institute for Advanced Neutron and X-Ray Science, Lund, Sweden; ^4^Biolfim-Research Center for Biointerfaces and Biomedical Science Department, Faculty of Health and Society, Malmö University, Malmö, Sweden; ^5^National Deuteration Facility, Australian Nuclear Science and Technology Organisation, Lucas Heights, NSW, Australia; ^6^Department of Chemical Engineering, Monash University, Clayton, VIC, Australia

**Keywords:** self-assembly, peptide structure, membrane protein, lipid cubic phase, small-angle scattering

## Abstract

This perspective describes advances in determining membrane protein structures in lipid bilayers using small-angle neutron scattering (SANS). Differentially labeled detergents with a homogeneous scattering length density facilitate contrast matching of detergent micelles; this has previously been used successfully to obtain the structures of membrane proteins. However, detergent micelles do not mimic the lipid bilayer environment of the cell membrane *in vivo*. Deuterated vesicles can be used to obtain the radius of gyration of membrane proteins, but protein-protein interference effects within the vesicles severely limits this method such that the protein structure cannot be modeled. We show herein that different membrane protein conformations can be distinguished within the lipid bilayer of the bicontinuous cubic phase using contrast-matching. Time-resolved studies performed using SANS illustrate the complex phase behavior in lyotropic liquid crystalline systems and emphasize the importance of this development. We believe that studying membrane protein structures and phase behavior in contrast-matched lipid bilayers will advance both biological and pharmaceutical applications of membrane-associated proteins, biosensors and food science.

## Introduction

Integral and peripheral membrane proteins play an important role in signal transduction, solute transport, energy conversion and charge separation in eukaryotic and prokaryotic cells (Gaur and Natekar, 2010). Such membrane proteins are also important drug targets, with more than half of all pharmaceutical compounds on the market targeting membrane proteins (Service, 2014). There are significant experimental challenges with determining membrane protein structures in their native state due to their limited solubility in water. Membrane protein structures should, therefore, ideally be investigated within a native lipid bilayer environment. Although some studies have used vesicles to investigate membrane proteins in a lipid bilayer environment, most studies to date have focused on using detergent micelles which do not effectively mimic the lipid bilayer. In this perspective we describe advances in membrane protein structure determination in detergent micelles as well as vesicles. These studies are compared with new data obtained using bicontinuous cubic phases.

Bicontinuous cubic phases, formed via lipid self-assembly, consist of a single lipid bilayer convoluted over three-dimensional space and subdividing space into two interpenetrating aqueous networks. The lipid bilayer adopts the shape of a theoretical surface of zero mean curvature, but with a continually varying Gaussian curvature which is everywhere non-positive (Luzzati et al., 1968). The three known bicontinuous cubic phases are based on the Schoen gyroid (G) minimal surface, the Schwartz primitive (P) minimal surface, and the Schwartz diamond (D) minimal surface, and are denoted ${\text{Q}}_{\text{II}}^{\text{G}}$, ${\text{Q}}_{\text{II}}^{\text{P}}$, and ${\text{Q}}_{\text{II}}^{\text{D}}$, respectively. The bulk bicontinuous cubic phases, which have the form of a viscous sticky gel, may be dispersed into sub-micron particles known as cubosomes, which must be sterically stabilized against flocculation via the addition of a polymer-based stabilizer, such as Pluronic F127 (Barriga et al., 2019; Fornasier et al., 2020). The elegant, bilayer-based, amphiphilic nanostructure of the cubic phase provides an ideal matrix for the encapsulation of transmembrane and membrane-associated proteins and peptides with a wide range of molecular masses (Conn and Drummond, 2013). Water-soluble proteins may also be encapsulated within the aqueous networks. The main applications of the bicontinuous cubic phases, including as drug delivery vehicles (Zhai et al., 2019), biosensors (Vallooran et al., 2016) and matrices for membrane protein crystallization (Caffrey, 2009), depend on their properties, such as the ability to encapsulate hydrophobic and hydrophilic moieties, large surface-area, biocompatibility, and retention of functionality for encapsulated proteins.

Encapsulation of a specific protein can impact the underlying cubic nanostructure depending on a range of factors including the charge on the protein and geometric mismatch between the protein and the aqueous channel diameter (for water soluble proteins) or bilayer thickness (for membrane-associated proteins) (Angelova et al., 2012; van 't Hag et al., 2016b, 2017b; Meikle et al., 2017). For water-soluble proteins or peptides, encapsulation may be quantified by separating the bulk cubic phase, or cubosomes, from the surrounding aqueous phase via centrifugal filtration. Loading efficiency may be calculated by using UV absorption to determine the peptide or protein concentration in the filtrate. For poorly water-soluble peptides, either the insoluble precipitate, or the loaded cubosomes, may be analyzed to determine the peptide loading. Encapsulation efficiencies of up to 90 mol% have been measured for smaller peptides, such as some antimicrobial peptides (Conn et al., 2011; Boge et al., 2019). In contrast, for larger proteins, such as GPCRs, the underlying cubic nanostructure may be completely disrupted at low protein concentrations of <3 mol% (Conn et al., 2010).

The lipid bilayer structure of the bicontinuous cubic phase can provide an ideal matrix for structure determination in a more biomimetic environment compared to detergent micelles. Neutron scattering with contrast-variation allows for the isolation of scattering from individual components within a complex system of protein, lipid(s), and solvent (water or buffer). The neutron scattering contrast can be varied over a wide range using the difference in neutron scattering length density (SLD) between hydrogen and deuterium. Isotopic substitution of this type usually has small but volume fraction defined effects on the phase diagram of lipid mesophases in water (Vandoolaeghe et al., 2009b; Bremer et al., 2017; Bryant et al., 2019). The effects for hydrogens which take part in hydrogen bonding, e.g., water (Takahashi and Jojiki, 2017) are more complex (Bryant et al., 2019) but may also account for small shifts in the phase boundaries. We describe how this has been used to study proteins in detergent micelles and vesicles and show that this has now been successfully applied to study peptide structures in contrast-matched lipid cubic phases. Advances in using SANS for time-resolved studies will also be discussed.

## Membrane Protein Structures in Lipid Membranes

### Detergents

Early attempts to study membrane proteins with contrast-matched detergent micelles were performed on rhodopsin in the 1970s (Osborne et al., 1978). Since that time, this approach has been used to study the structure of other membrane proteins (Breyton et al., 2013; Naing et al., 2018), however, these studies tended to focus on the radius of gyration and oligomeric state of the proteins. More recently, structural work on membrane proteins showed how the use of differentially labeled detergents with a homogeneous scattering length density allowed for superior contrast-matching of the detergent micelle; consequently, the scattering curves were a more faithful representation of the isolated membrane protein (Midtgaard et al., 2018). At the same time, approaches are being developed to model the protein structure and the detergent micelles from SANS or SAXS (Pérez and Koutsioubas, 2015; Koutsioubas, 2017), but the impact remains modest, largely due to the difficulties involved in working with membrane proteins. Additionally, as detergent micelles do not mimic the lipid bilayer environment of the cell membrane *in vivo*, the structure and function of membrane proteins can be significantly affected in a detergent environment (Bayburt and Sligar, 2010; Breyton et al., 2013).

### Vesicles

SANS has also been used to study membrane protein assemblies and, in particular, oligomerization in liposomes (Hunt et al., 1997). In this case, liposome polydispersity must be accounted for which is a significant challenge. Fitting of a membrane associated protein with vesicles was, for example, modeled using five parameters: liposome radius, lipid thickness, lipid bilayer SLD, protein thickness and protein SLD. Relative differences in conformation could be detected using this method (Satsoura et al., 2012; Heberle et al., 2013). In another study, results were mainly focused on changes in the lipid bilayer thickness and not the protein structures (Gilbert et al., 1999). In recent years there has been significant progress in modeling complex phase-behavior, such as the formation of lipid rafts in lipid-only vesicles using SAXS and SANS (Marquardt et al., 2015; Heberle and Pabst, 2017).

Protein-protein interference effects due to the structure factor caused by the large number of protein monomers per vesicle are a significant limitation when studying membrane proteins reconstituted in vesicles. For bacteriorhodopsin the radius of gyration was obtained in contrast-matched vesicles of dimyristoylphosphatidylcholine (DMPC-d_67_-h_5_). However, the study indicated that only a limited q-region (0.01–0.05 Å^−1^) was free from interference effects (Hunt et al., 1997). While the limited q-range allowed for determination of the radius of gyration, it was not possible to obtain the detailed protein conformation which relates to its function. *Ab initio* modeling of membrane protein structures in a lipid bilayer membrane has had limited success to date (Breyton et al., 2013; Skar-Gislinge et al., 2015).

### Cubic Phases

Membrane protein encapsulation can also be studied using the lipid self-assembly bulk bicontinuous cubic phase, which has a significantly higher surface area to volume ratio compared to vesicles. This affords the significant advantage that large amounts of protein can be encapsulated, increasing the neutron scattering signal. To prepare the bicontinuous cubic phases, lipid (50–80% v/v) is typically mixed with membrane protein solution (50–20% v/v) in detergent. For this preparation method, there is no point where the protein is completely separated from the detergent micelle or lipid bilayer (van 't Hag et al., 2017a).

Herein, we present new data where hydrophobic peptides are directly mixed with lipid without the need to use detergents. We have recently shown that deuterated lipids can be used to contrast match the lipid membrane to D_2_O, in order to isolate the scattering from the peptides. This can be used to retrieve the Bragg peaks from the cubic phase geometry due to the scattering of the homogeneously encapsulated peptides in the contrast-matched membrane (van 't Hag et al., 2016a, 2019). For gramicidin A in the lipid cubic phase of monoolein (MO), an increase in Bragg peak intensity was found with increasing peptide concentration (van 't Hag et al., 2019). In contrast, the cubic phase formed by the branched chain lipid phytanoyl monoethanolamine (PE) showed Bragg peaks at low peptide concentrations, but limited WALP21 and WALPS53 encapsulation at high concentrations (van 't Hag et al., 2016a,b). This can be explained by the significantly higher lateral bilayer pressure in the case of PE bilayers and illustrates the importance of the physicochemical properties of the membrane.

**Figure 1:**
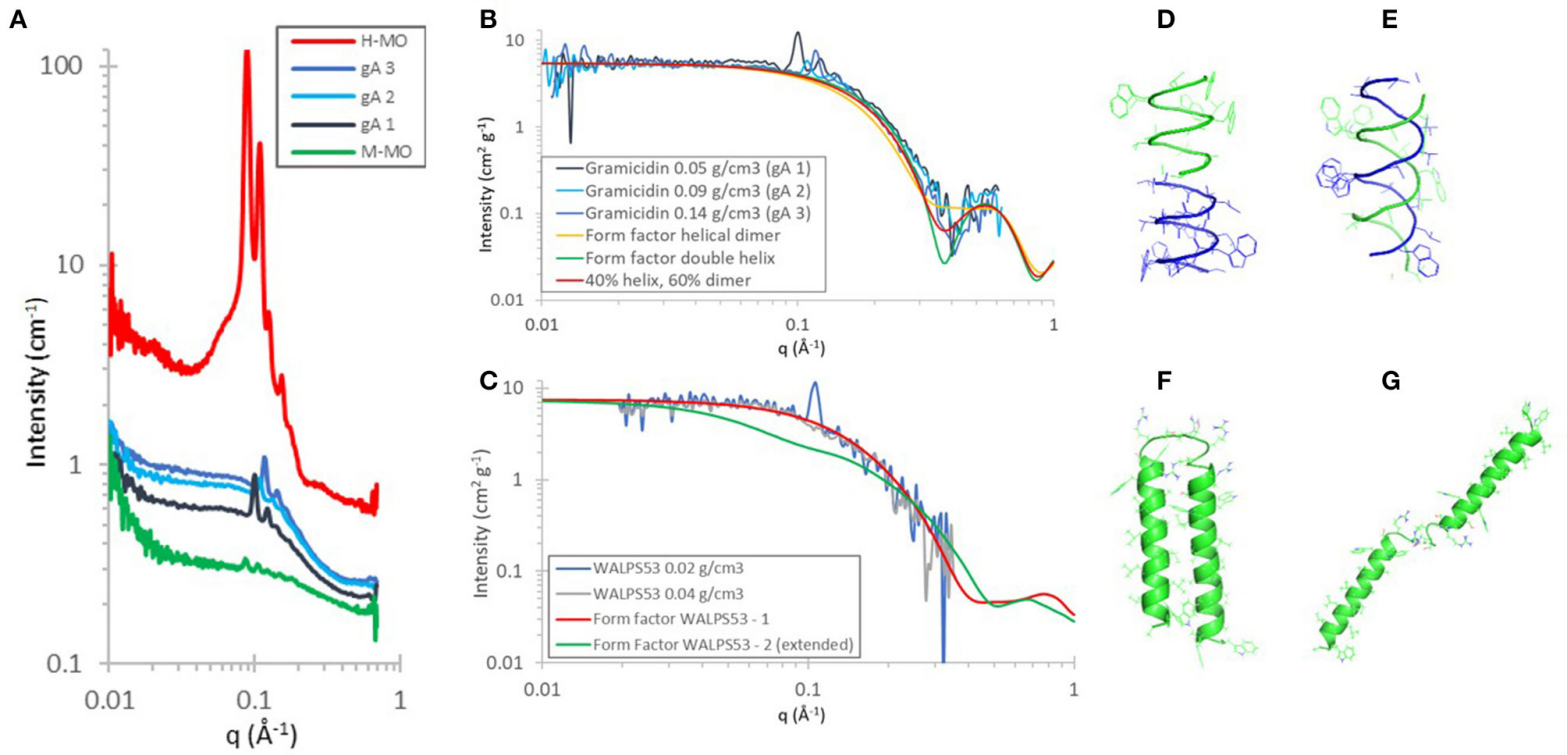
**(A–C)** SANS data showing H-MO and peptide encapsulation in 92% D-MO/8% H-MO (M-MO) at a range of peptide concentrations at 20°C in D_2_O. **(A,B)** Gramicidin A (1.9 kDa) concentrations reflect 1.6, 3.1, and 5.3 mol% with respect to MO and at 40% v/v D_2_O: diamond cubic ${\text{Q}}_{\text{II}}^{\text{D}}$ phase. **(C)** WALPS53 (5.9 kDa) at 0.2 and 0.4 mol% with respect to MO and at 35% v/v D_2_O: gyroid cubic ${\text{Q}}_{\text{II}}^{\text{G}}$ phase. The SANS data was treated by first subtracting the MO cubic phase as background, then scaled to peptide concentration, and then a further incoherent background was subtracted. The calculated form factors were obtained using CRYSON. **(D–G)** were created using PyMOL and show cartoon representations of the secondary structures with side-chains shown as lines. They were obtained using PDB ID **(D)** 1MAG: helical dimer and **(E)** 1BDW: double helix. **(F)** WALPS53–1 dimer as generated using the iTasser server (Yang et al., 2015) and **(G)** WALPS53–2 (extended version of WALPS53–1).

Herein, we extend this work by investigating the peptide structures of Gramicidin A (gA) and WALPS53 in the contrast-matched cubic Q_II_ phase of MO (M-MO) (Figure 1). gA can appear in two different conformations in a lipid bilayer: as a helical dimer (PDF ID: 1MAG, Figure 1D) and/or as a double helix (PDB ID: 1BDW, Figure 1E). The time-of-flight SANS data, as collected during a single measurement of each sample and scaled to peptide concentration, was in excellent quantitative agreement with the gA form factor of a mixture of helical dimer and double helix (Figures 1A,B). An approximately equal contribution of each structure is in agreement with what was found using circular dichroism spectroscopy of gA in the diamond cubic ${\text{Q}}_{\text{II}}^{\text{D}}$ phase of MO (Meikle et al., 2017). The simulated form factor data was obtained using CRYSON (v 2.7) (Svergun et al., 1998), using the gA structures (PDB ID: 1MAG and PDB ID: 1BSW), assuming a D_2_O solvent concentration of 100%, and setting the contrast of the solvation shell to 0. The form factors from CRYSON were multiplied by *N*_A_/*M*_w_ × 10^−24^, permitting direct comparison with SANS data on an absolute scale normalized by peptide concentration.

The model peptide WALPS53 consists of two transmembrane α-helices [amino acid sequence GWW-(LA)_7_L-WWA] bound by a short hydrophilic domain [amino acid sequence S(RS)_5_]. In the cubic phase of MO with a water channel diameter of ~50 Å, the WALPS53 hydrophilic domain, with a length of ~46 Å (based on a distance of 3.5 Å between α-carbons) (van 't Hag et al., 2016b), would be able to bridge the water channels based on geometrical considerations. The two peptide conformations investigated are therefore (i) one where the transmembrane α-helices are present as a dimer (Figure 1F) and (ii) another where the structure is fully extended (Figure 1G). A model structure of the WALPS53 dimer was generated using the iTasser server (Yang et al., 2015), while the extended structure was generated from that dimer by manually rotating the C-terminal helix (S30-A53) around residue R29 by an angle of ~180° using PyMol (Version 1.2r3pre, Schrödinger, LLC). This simple two state model allows for comparison of the two extreme conformations: extended vs. compact. We note, however, that in the extended conformation the two helices are expected to be able to move freely around the linker region. The form factors for the fully extended and compact structures were calculated for each structure as described above for gA. The SANS data with two different peptide concentrations showed that the peptide is present as a dimer in the gyroid cubic ${\text{Q}}_{\text{II}}^{\text{G}}$ phase at 0.2 and 0.4 mol% and 35% v/v D_2_O. The data for WALPS53 are noisier than data for gA due to the significantly lower peptide concentrations which also resulted in a narrower available q-range after subtraction. Nevertheless, the SANS data shows good quantitative agreement with the simulated form factor of the dimeric form of the peptide as predicted by the iTasser server.

## Dispersed Cubic Phases for Therapeutic Protein Delivery

It was recently shown that the internal structure of cubosomes (dispersed cubic phases) can also be contrast-matched (Yepuri et al., 2019). These nanoparticles can be used for the delivery of therapeutic and antimicrobial peptides, imaging agents and hydrophobic drugs (Mulet et al., 2013; Zhai et al., 2015). Polymeric stabilizers, such as Pluronic F127 and Tween 80, used to prevent aggregation of the cubosomes are known to affect the internal cubic phase nanostructure. For phytantriol cubosomes, deuterated phytantriol was used to prove that Tween 80 was distributed throughout the internal lipid bilayer network and not just on the surface, which led to a phase transition to the more swollen and less curved primitive cubic ${\text{Q}}_{\text{II}}^{\text{P}}$ phase (Yepuri et al., 2019). This demonstrates that SANS with contrast-matched cubic phases can also be used to study dispersed cubic phase systems, and opens up the possibility to study release of bioactives from cubosome nanoparticles using this technique.

## Time-Resolved Studies on Multi-Component Systems Using Sans

The non-destructive nature of the neutron beam makes SANS suitable for *in situ* kinetic studies. We describe how this has been used to study lyotropic liquid crystalline systems to date, and could be used in the future, to study the kinetics of hybrid protein-lipid materials. Kinetic studies are of fundamental importance for understanding what happens during delivery and digestion of therapeutic proteins (Conn and Drummond, 2013; Mulet et al., 2013), enzymatic reactions in lipid phases for use as biosensors and biofuel cells (Nazaruk et al., 2008; Vallooran et al., 2016), and during *in meso* crystallization of membrane proteins for high-resolution structure determination (Cherezov, 2011; van 't Hag et al., 2014; van 't Hag et al., 2019; Caffrey, 2015; Zabara et al., 2017).

SAXS and SANS have commonly been used to study the phase transitions and geometric pathways between lyotropic liquid crystalline phases formed by a single amphiphile/surfactant in water (Hamley et al., 1998; Angelov et al., 2007; Vandoolaeghe et al., 2009a; Squires et al., 2015; van 't Hag et al., 2017b), whereby X-ray studies have a much higher time resolution but are limited by radiation damage and a single contrast. In 2000 it was reported that a significant increase in the neutron flux and detector size enabled time-resolved studies of relatively fast surfactant phase transitions in solution, elucidating the transition from micelles to vesicles as well as nucleation and growth of oil droplets with measurement times of 30 s (Egelhaaf et al., 2000). Additionally, hydrogels, polymer systems, emulsions, and surfactant lamellar phases are examples of systems that have been studied under flow, using Rheo-SANS. Bulk surfactant lamellar phases were suggested to form multi-lamellar vesicles under shear via intermediate cylinders or buckling. Currently most Rheo-SANS measurements focus on a time average and the ensemble structure formed by a single amphiphile (Eberle and Porcar, 2012). It was suggested that studying transient structural reorientation of complex fluids requires powerful neutron sources and techniques, but that multicomponent systems and contrast-matching will become invaluable for understanding the relation between the rheological properties and membrane structure by using new sources, such as the European Spallation Source and new techniques including examination of steady perturbed states (Glinka et al., 2020).

Angelov et al. used a combination of SANS and SAXS to show that the cubic phase behavior of the ternary system consisting of MO, water and octyl glucoside (OG) undergoes phase separation. At low temperatures the swollen cubic phases were found to have OG encapsulated within the lipid bilayer. In contrast, when heated to higher temperatures the OG molecules were suggested to be released into the aqueous water channels (Angelov et al., 2007; Angelova et al., 2011). Additionally, using SAXS the protein neurotrophin was found to impact the curvature of MO-eicosapentaenoic-water cubic phases within milliseconds, leading to a hydrated mixed ${\text{Q}}_{\text{II}}^{\text{D}}$/${\text{Q}}_{\text{II}}^{\text{G}}$ shell connected to a lamellar core phase (Angelov et al., 2014). Multi-phase liquid-crystalline nanoparticles with the cubic phase forming lipid MO have also been observed in a ternary mixture with capric acid and water (Killian, 2003). Recently, the complex phase behavior resulting from the interactions of silica nanoparticles with a ternary water-oil system was extensively studied using SANS with contrast-variation. Nanoparticles were found to aggregate at domain boundaries which led to a change in the liquid crystalline domain size (Marlow et al., 2019). In the case of triphilic star-polyphiles, hybrid double chain surfactants with a hydrocarbon and fluorocarbon chain, contrast-variation SANS was successfully used to show that the membrane was fully de-mixed into hydrocarbon and fluorocarbon domains in the hexagonal phase, demonstrating more complex phase behavior than that found in amphiphiles (de Campo et al., 2011).

Using the contrast-matched MO cubic phase we were able to follow the *in meso* crystallization of the biologically relevant transmembrane peptides glycophorin A (GpA) (Trenker et al., 2015) and DAP12 (Knoblich et al., 2015) using SANS. Peptide crystal growth was directly related to a significant decrease in form factor scattering intensity of the peptides. For DAP12 a transient fluid lamellar L_α_ phase was observed. In contrast, for GpA the lamellar crystalline L_c_ phase of the peptide single crystals was observed. This suggested that the specific mechanism is peptide and protein dependent (van 't Hag et al., 2019). This was the first time *in meso* crystallization was studied from the protein-eye perspective as previous studies using SAXS and SANS without contrast-matching focused, by necessity, on the lipid phase (Efremov et al., 2005; van 't Hag et al., 2016c; Zabara et al., 2017).

## Discussion

We have described current progress on the use of detergent micelles and vesicles to determine membrane protein structures. To date, neither of these environments have proved wholly successful in determining membrane protein conformations in a lipid bilayer environment. While membrane protein studies with phospholipid nanodiscs have recently gained a lot of attention (Bayburt and Sligar, 2010; Skar-Gislinge and Arleth, 2011) we note that they face significant challenges with regards to contrast-matching, mainly due to the use of an encircling helical protein belt that keeps the nanodiscs together. The new data presented herein, to the best of our knowledge, represents the first time that specific membrane protein conformations were determined in contrast-matched bicontinuous cubic phases using SANS. This is a promising approach for studying membrane proteins that are too small for structural studies with Cryo-EM (<100 kDa) (Cheng, 2018) and too large for NMR (>25 kDa) (Liang and Tamm, 2016).

We suggest that the use of contrast-matched cubic phases, as presented herein, is also suitable for investigating the structures of large and complex membrane proteins. Membrane proteins may be reconstituted directly into the cubic phase from a solution with deuterated detergents. Alternatively, the use of 100% D-MO (rather than 92% D-MO and 8% H-MO as presented herein) and H-detergent will also lead to contrast-matched cubic phases. Furthermore, swollen cubic phases could be employed for membrane proteins with a large hydrophilic domain (Zhai et al., 2020). The complex phase behavior in lyotropic liquid crystalline systems is evident in time-resolved studies and emphasize the importance of this development.

The use of SANS with contrast-matching has the potential to further advance our understanding of encapsulated proteins and other additives in lyotropic lipid cubic phases. Many questions remain, such as do large amphiphilic proteins encapsulate within the cubic phase water channels or in the cubic phase domain boundaries? How do protein molecules from several cubic phase domains feed the protein crystals during *in meso* crystallization? How do the lipid bilayer physicochemical properties affect protein structures (Frewein et al., 2016)? How can we improve the use of lipid nanoparticles with therapeutic peptides for oral delivery by studying digestion of the protein-lipid materials? Therapeutic peptides have shown to be a promising treatment for cancer, viral infections as well as multi-drug resistant bacterial infections (Walsh, 2014). However, their effective delivery remains a challenge.

Additionally, protein and peptide interactions with the cell membrane are of significant importance for pharmaceutical applications with more than half of all drugs on the market targeting membrane proteins (Service, 2014). Biological cell membranes consist of hundreds of different lipids and display complex phase behavior (Sarkar et al., 2019). We believe that studying membrane protein structures and phase behavior in contrast-matched lipid bilayers will advance biological and pharmaceutical applications, biosensors and food science. Combined with recent advances in chemical and biological deuteration, neutron scattering technology and instrumentation we expect a bright future for this research field.

## Materials and Methods

### Materials

H-MO, gramicidin from *Bacillus aneurinolyticus*, hexafluoroisopropanol (HFIP) and methanol-d1 (MeOD) was purchased from Sigma Aldrich (St Louis, MO). Hamilton syringes were obtained from Hamilton Company (Reno, NV) and syringe couplers from TTP Labtech (Cambridge, MA). WALPS53 with >95% purify was purchased from CS Bio, Inc. (Menlo Park, CA, USA) and mass and purity were confirmed (van 't Hag et al., 2016b). The synthesis of D-MO was described in a previous publication (van 't Hag et al., 2019).

### Sample Preparation

Initially, the exchangeable hydrogens of H-MO and D-MO were exchanged for deuterium using MeOD. Homogeneous peptide/lipid mixtures were obtained by co-dissolving the peptide with the appropriate H/D-MO mixture: gA with H/D-MO was dissolved in 260 μL MeOD or WALPS53 was dissolved in 100 μL HFIP, and then mixed with H/D-MO in 160 μL MeOD. While in the gA samples the hydrogens remained fully exchanged, the co-solvation process for WALP53 led to an exchangeable H/D ratio of ~1:4. Most of the solvent was evaporated using a stream of N_2_, followed by freezing in liquid N_2_ followed by lyophilizing overnight. D_2_O was loaded into the first 100 μL Hamilton syringe, the second syringe was loaded with the peptide/lipid mixture after it was molten at 40°C. Samples were mixed at 35% v/v or 40% v/v D_2_O.

### Small-Angle Neutron Scattering (SANS)

SANS measurements were performed on BILBY at the ANSTO OPAL reactor in time-of-flight mode using neutrons with wavelengths between 3 and 12 Å (Sokolova et al., 2019; van 't Hag et al., 2019). The sample detector distances were 1.8 m (horizontal curtains), 2.8 m (vertical curtains) and 6.0 m (rear). The collimation length was 6.7 mm and a sample aperture of 7.5 mm was used. Using the Mantid software (Arnold et al., 2014) the data was put on absolute scale using a direct beam measurement and corrected for scattering contributions from the background.

## Data Availability Statement

The raw data supporting the conclusions of this article will be made available by the authors, without undue reservation.

## Author Contributions

CC performed SANS measurements, assisted with design of the study, and drafting the manuscript. LC developed and facilitated TOF-SANS measurements and data analysis protocols. AW performed the peptide structure modeling and form factor simulations. CG provided the critical commentary on the results and manuscript. AK-H performed the synthesis and characterization of deuterated MO. LH designed the study, analyzed the data, and drafted the manuscript. All authors contributed to editing the paper and gave final approval for publication.

## Conflict of Interest

The authors declare that the research was conducted in the absence of any commercial or financial relationships that could be construed as a potential conflict of interest.
